# Vitamin D3-Induced Tolerogenic Dendritic Cells Modulate the Transcriptomic Profile of T CD4^+^ Cells Towards a Functional Hyporesponsiveness

**DOI:** 10.3389/fimmu.2020.599623

**Published:** 2021-01-20

**Authors:** Juan Navarro-Barriuso, María José Mansilla, Bibiana Quirant-Sánchez, Aina Teniente-Serra, Cristina Ramo-Tello, Eva M. Martínez-Cáceres

**Affiliations:** ^1^ Division of Immunology, LCMN, Germans Trias i Pujol University Hospital and Research Institute, Barcelona, Spain; ^2^ Department of Cellular Biology, Physiology and Immunology, Universitat Autònoma de Barcelona, Barcelona, Spain; ^3^ Multiple Sclerosis Unit, Department of Neurosciences, Germans Trias i Pujol University Hospital, Barcelona, Spain

**Keywords:** tolerogenic dendritic cells, immune tolerance, T cells, antigen-specific response, transcriptomic study

## Abstract

The use of autologous tolerogenic dendritic cells (tolDC) has become a promising alternative for the treatment of autoimmune diseases. Among the different strategies available, the use of vitamin D3 for the generation of tolDC (vitD3-tolDC) constitutes one of the most robust approaches due to their immune regulatory properties, which are currently being tested in clinical trials. However, the mechanisms that vitD3-tolDC trigger for the induction of tolerance remain elusive. For this reason, we performed a full phenotypical, functional, and transcriptomic characterization of T cells upon their interaction with autologous, antigen-specific vitD3-tolDC. We observed a strong antigen-specific reduction of T cell proliferation, combined with a decrease in the relative prevalence of T_H_1 subpopulations and IFN-*γ* production. The analysis of the transcriptomic profile of T CD4^+^ cells evidenced a significant down-modulation of genes involved in cell cycle and cell response to mainly pro-inflammatory immune-related stimuli, highlighting the role of *JUNB* gene as a potential biomarker of these processes. Consequently, our results show the induction of a strong antigen-specific hyporesponsiveness combined with a reduction on the T_H_1 immune profile of T cells upon their interaction with vitD3-tolDC, which manifests the regulatory properties of these cells and, therefore, their therapeutic potential in the clinic.

## Introduction

In the last years, tolerogenic dendritic cells (tolDC) have become one of the most promising alternatives for the treatment of autoimmune diseases, such as multiple sclerosis (MS), rheumatoid arthritis, or type 1 diabetes. In fact, several Phase I clinical trials have already finished or are currently ongoing, with positive results regarding the safety and the tolerability of this therapeutic cell-based approach (1). In general, tolDC are commonly defined as a stable and semi-mature subset of dendritic cells (DC), between antigen-capturing immature DC (iDC) and immunogenic mature DC (mDC)—characterized by their increased expression of MHC class II and co-stimulatory molecules. But most importantly, tolDC are presumably capable to induce immune tolerance towards the peptides these cells are presenting, in an antigen-specific manner (2–5).

TolDC can be generated *in vitro* from peripheral blood monocytes. In the last years, a wide variety of protocols for their production have been reported, ranging from the use of different drugs and chemical agents to genetic engineering techniques (6, 7). In this regard, the use of 1,25-dyhydroxyvitamin D3, the active form of vitamin D3, constitutes one of the most widely studied approaches for the differentiation of tolDC. Briefly, vitamin D3-induced tolDC (vitD3-tolDC) are thought to develop their regulatory properties through a semi-mature profile, their ability to inhibit or reduce T cell responses, and a switch of the immune response towards a T_H_2 profile (8–18). Furthermore, vitD3-tolDC are characterized by a reduced NF-*κ*B-mediated activity and an increase of mTOR-mediated glucose metabolism (10, 19).

Even though tolDC—and vitD3-tolDC in particular—have been characterized with a developing knowledge over their metabolism, molecular mechanisms, and functional pathways, the specific effect of these cells over the rest of the immune-related components still remains elusive. It is known that tolDC can usually induce either anergy, hyporesponsiveness or depletion over activated T cells, as well as regulatory T cell (Treg) differentiation (20). However, to our knowledge, so far only one study has focused its attention on the actual processes that autologous T cells might be undergoing upon tolDC interaction—reporting an induction of hyporesponsiveness of CD4^+^ memory and naïve T cells towards antigen-specific stimulation mediated by dexamethasone-induced tolDC (21)—but neither at the transcriptomic level nor with vitD3-tolDC in particular.

In previous studies, our group has already extensively characterized vitD3-tolDC phenotypically, functionally, and transcriptomically, evidencing the regulatory potential of these cells both *in vitro* and *in vivo* in the animal model of MS, experimental autoimmune encephalomyelitis (EAE) (13, 16, 22–24). Consequently, we wanted to take one step further for the elucidation of the mechanisms of immune tolerance induction of vitD3-tolDC. With that aim, here we present a full phenotypical, functional, and transcriptomic characterization of T CD4^+^ cells after their interaction with autologous vitD3-tolDC loaded with tetanus toxin (TT), in order to study the antigen-specific effect mediated by these cells compared to TT-loaded immunogenic mDC. The purpose of this study is to identify one or several potential biomarkers of the immune modulation developed by vitD3-tolDC over T cells, which could constitute an interesting tool for the monitoring of patients treated with these cells in clinical trials, and the understanding of the mechanisms of tolerance induction.

## Material and Methods

### Sample Collection

Buffy coat samples from 16 randomized healthy controls were obtained from the *Banc de Sang i Teixits* (Barcelona, Spain), according to the institutional Standard Operating Procedures for blood donation, including a signed informed consent. In parallel, whole blood samples from 12 different healthy donors were collected by standard venipuncture in lithium heparin tubes for the allogeneic functional assays (see below).

### Monocyte Isolation

Healthy donor buffy coat samples were processed first depleting CD3^+^ cells using the RoseetteSep® Human Monocyte Enrichment Kit (StemCell Technologies, Vancouver, Canada) prior to a density gradient separation using ficoll-hypaque (Rafer, Zaragoza, Spain). Afterwards, CD14^+^ cells were isolated using the EasySep® Human CD14 Positive Selection Kit (StemCell), according to the manufacturer’s instructions. Cell viability was determined using 7-amino-actinomycin D (7-AAD) (BD Biosciences, Franklin Lakes, NK, USA) and phycoerythrin (PE)-conjugated annexin V (Immunotools, Friesoythe, Germany) staining for 20 min at 4°C, protected from light, and cell counts were quantified simultaneously using PerfectCount beads (Cytognos, Salamanca, Spain). Samples were acquired on a FACSCanto II flow cytometer (BD Biosciences), and monocyte purity was determined using forward and side scatter gating strategies on FACSDiva software (BD Biosciences).

### TT-Loaded DC Cultures

The protocol for the generation of antigen-loaded tolDC was adapted from a previous study (25). Briefly, isolated monocytes were cultured for 6 days in 24-well plates at 37°C at a density of 1 × 10^6^ cells/ml in X-VIVO 15 medium, in the presence of 400 U/ml granulocyte-macrophage colony-stimulating factor (GM-CSF) and 500 U/ml IL-4 (both from Peprotech, London, UK). The whole volume of medium and cytokines was replenished on day 4. If no further treatment was performed, monocytes were differentiated into iDC. For the generation of mDC, we further added a maturation cocktail, containing 1,000 U/ml IL-1β, 1,000 U/ml TNF-α (both from Peprotech) and 1 µM prostaglandin E2 (PGE2) (Pfizer, New York, NY, USA) on day 4. Finally, in addition to the maturation cocktail, we added 1 nM vitamin D3 (Calcijex, Abbott, Chicago, IL, USA) on days 0 and 4 for the differentiation of vitD3-tolDC. For the generation of TT-loaded mDC (mDC-TT) and TT-loaded vitD3-tolDC (vitD3-tolDC-TT) as antigen-specific experimental conditions, 0.1 µg/ml of the whole TT protein (Sigma-Aldrich, St. Louis, MO, USA) were added to the mDC and vitD3-tolDC cultures on day 3, 18 h before the addition of the maturation stimulus, while still in an immature status. On day 6, cells were harvested after an accutase (Invitrogen, Carlsbad, CA, USA) detaching treatment for 30 min, and washed twice. As shown above, cell counts and viability were determined by flow cytometry.

### Autologous PBMC Isolation, Co-Culture, and Sorting

For the isolation of autologous PBMC, 3 ml of the buffy coat samples from each healthy donor was processed using a ficoll-hypaque density gradient separation and washed twice. Afterwards, cells were counted by flow cytometry, as described above, and plated in round-bottom 96-well plates at a density of 1 × 10^6^ cells/ml in RPMI medium supplemented with 10% heat-inactivated fetal bovine serum, 2 mM L-glutamine (Lonza, Basel, Switzerland), 100 U/ml penicillin (Reig Jofre, Sant Joan Despí, Spain) and 100 µg/ml streptomycin (Normon, Tres Cantos, Spain). The plates were then incubated for 6 days at 37°C in a 5% CO_2_ atmosphere. Afterwards, cells were harvested, and cell counts and viability were determined by flow cytometry.

Subsequently, an antigen-specific proliferation experimental setup was performed in 96-well round-bottom plates with co-cultures of 10^5^ autologous PBMC and 5,000 either mDC-TT or vitD3-tolDC-TT (1:20 ratio) in a final volume of 200 µl of supplemented RPMI medium. For each condition, 48 replicates were performed. Cells were then incubated for 5 days at 37°C in a 5% CO_2_ atmosphere. Afterwards, cells were harvested and the whole volume of each cell suspension was incubated for 20 min, protected from light, with the adequate amounts of monoclonal antibodies anti-CD3 Violet 450 (V450) and anti-CD4 PerCP-Cyanine dye (Cy)5.5. Finally, cells were washed and the whole CD3^+^CD4^+^ cell subpopulation was isolated and counted using a FACSAria II cell sorter (BD Biosciences). Finally, dry pellets of mDC-TT-conditioned CD3^+^CD4^+^ cells (mDC-Tcell) and vitD3-tolDC-TT-conditioned CD3^+^CD4^+^ cells (vitD3-Tcell) were obtained by centrifugation and stored at −80°C.

### Phenotype Characterization of DC and Autologous PBMC

Surface expression of CD11c, CD14, CD83, CD86 and HLA-DR in iDC, mDC, mDC-TT, vitD3-tolDC and vitD3-tolDC-TT was determined by flow cytometry. In each case, DC suspensions were incubated for 20 min, protected from light, with the appropriate amounts of monoclonal antibodies anti-: CD11c PE-Cy7, CD14 V450, CD83 allophycocyanin (APC), CD86 fluorescein isothiocyanate (FITC) and HLA-DR Violet 500 (V500) (all of them from BD Biosciences). Subsequently, at least 10,000 CD11c^+^ events of each condition were acquired using a FACSCanto II flow cytometer and analyzed using FACSDiva software.

For the phenotypical characterization of mDC-Tcell and vitD3-Tcell, cell suspensions of these conditions were incubated for 20 min with the adequate amounts of monoclonal antibodies indicated below. Afterwards, samples were washed twice and acquired on a LSRFortessa flow cytometer, setting the stopping gate at 300,000 peripheral blood mononuclear cells. The definition of each peripheral blood mononuclear cell subpopulation was determined as specified in **Supplementary Table 1**, using several combinations of the following monoclonal antibodies anti-: CXCR3 AlexaFluor (AF)488, CD4 PerCP-Cy5.5, CCR7 PE, CD45RA PE-Cy7, CD38 APC, CD45 AF700, CD8 APC-H7, CD3 V450, HLA-DR V500, CCR6 Brilliant Violet (BV) 605, CD25 PE, CCR4 PE-Cy7, CD127 AF647, CD45RO APC-H7, CD49b FITC and LAG-3 PE (BD Biosciences). Results were analyzed with FACSDiva software (BD Biosciences). Forward and side scatter gating strategy was used in order to select the desired lymphocyte subpopulations, and their relative percentages were analyzed for each cell subset.

### Allogeneic and Autologous Cell Proliferation Assays

For the determination of the reactivity of PBMC from each donor against TT, 2 × 10^5^ PBMC were plated in 96-well round bottom plates at day 0 of each culture in supplemented RPMI medium containing 0.1 µg/ml TT. As control conditions, the same number of cells was cultured with either supplemented RPMI medium only (negative control) or 50 ng/ml phorbol 12-myristate-13-acetate (PMA) and 500 ng/ml ionomycin (positive control). Ten replicates were performed for the negative control and the condition of analysis, and six replicates for the positive control. Cells were then cultured for 5 days at 37°C in a 5% CO_2_ atmosphere. Afterwards, 1 µCi [^3^H]-thymidine (PerkinElmer, Waltham, MA, USA) was added to each well, and the plate was incubated for further 18 h under the same conditions. Cells were then collected using a HARVESTER96 2M cell harvester (Tomtec Inc, Hamdem, CT, USA) and read on a 1450 MicroBeta TriLux liquid scintillation counter (Wallac, Turku, Finland). Donors were considered positive for TT reactivity when the counts per minute (cpm) of at least five replicates from the condition of analysis were over the mean plus two times the standard deviation (SD) of the negative control.

For the isolation of allogeneic PBMC, whole blood samples of different healthy donors were processed by ficoll-hypaque density gradient separation. Cells were washed twice, and afterwards, their absolute number and viability was determined as shown above. Subsequently, 10^5^ either allogeneic or autologous viable PBMC were co-cultured with 5,000 either iDC, mDC, mDC-TT, vitD3-tolDC or vitD3-tolDC-TT (1:20 ratio) in 96-well round bottom plates, in a total volume of 200 µl of supplemented RPMI medium. Again, as negative and positive controls, either supplemented RPMI medium or a mix of 50 ng/ml PMA and 500 ng/ml ionomycin was used, respectively. Six replicates of each condition were performed. Cells were then plated for 4 days at 37°C in a 5% CO_2_ atmosphere, and afterwards, 1 µCi [^3^H]-thymidine was added to each well, and the plates were incubated, harvested, and read as described above.

### Cytokine and Soluble Protein Production

The production of granzyme B (GZMB), as well as of IL-1β, IL-6, IL-10, IFN-*γ*, IL-12p70 and TNF-α cytokines, was quantified in the supernatants of mDC-TT and vitD3-tolDC-TT with autologous PBMC co-cultures, using the Human Soluble Protein CBA Flex Set (BD biosciences) according to manufacturer’s instructions. Samples were acquired on an LSR Fortessa flow cytometer (BD Biosciences), and the results were analyzed using FACSDiva software. The production of TGF-*β* was determined using the Human/Mouse TGF beta 1 Uncoated ELISA kit (Invitrogen) in 100 µl of the co-culture supernatants after sample activation with HCl 1N, following the manufacturer’s instructions. The optical density of each well was measured at *λ* = 450 nm, and the optical density at *λ* = 570 nm was then subtracted as background signal, using a Varioskan Flash Multimode Reader (Thermo Fisher Scientific, Waltham, MA, USA).

### RNA Extraction and RNA-seq Analysis

Total RNA of autologous mDC-Tcell and vitD3-Tcell samples was isolated using the automated Maxwell 16 LEV simplyRNA Purification Kit (Promega Biotech, Madison, WI, USA), including a DNAse I digestion step, according to manufacturer’s instructions. Samples were quantified using a Nanodrop ND-1000 spectrophotometer (Thermo Fisher Scientific), and subsequently stored at −80°C in RNAse-free tubes. RNA integrity number (RIN) was determined in an Agilent BioAnalyzer with the RNA6000 Pico assay (Agilent Technologies, Santa Clara, CA, USA). Afterwards, the sequencing libraries were prepared using the TruSeq Stranded Total RNA Sample Preparation kit (Illumina, San Diego, CA, USA) with 200 ng of total RNA per sample as input. Paired-end sequencing (2 × 75 bp) was then performed on a HiSeq-2500 instrument (Illumina). Reads were quality trimmed and adapters removed using Trimmomatic V0.30. TopHat software v2.1.0 was used to map RNA-seq reads to the human reference genome (Ensembl release 78) (26). FeatureCounts function was used to assign reads to genomic features focusing on RNA biotypes. A matrix with summarized raw counts of reads assigned through mapping to high confidence protein coding genes only was generated (“golden” annotation label), corresponding to stable and unlikely to change transcripts from the Consensus CDS (CCDS) Project. Data exploration results from hierarchical clustering and principal component analysis (PCA) in R software were used to exclude any outliers and assess sample similarities based on global gene expression patterns, and to guide the modeling design to be used for subsequent analyses.

### Differential Gene Expression Analysis

Transcriptional changes at the gene level between mDC-Tcell and vitD3-Tcell were assessed using the Bioconductor DeSeq2 package in R (27). A paired sample comparison design, factoring in inter-individual differences, was applied. The results were considered statistically significant with an adjusted p-value (padj) < 0.05. We set a 20% fold change (FC) cutoff as the threshold for relevant biological effects (|FC| > 1.2).

### Gene Ontology Enrichment Analysis

Unranked lists of the significant differentially upregulated (FC > 1.2 and padj < 0.05) and downregulated genes (FC < −1.2 and padj < 0.05) were tested for enrichment in Gene Ontology (GO) functional categories using the GOrilla web tool, applying the default settings for comparison to the background list of genes found in the dataset (28). We tested for enrichment in three types of GO categories: “biological process” (GOPROCESS), “molecular function” (GOFUNCTION), and “cellular component” (GOCOMPONENT). Enrichment score (ES) was defined as ES = (b/n)/(B/N), where “N” is the total number of genes in the background list, “B” is the total number of genes in N associated with a specific GO term, “n” is the number of differentially expressed genes being tested for enrichment and “b” is the number of n intersecting with B. Enrichment p-value is computed according to the hypergeometric (HG) model. False discovery rate (FDR) q-value is the Benjamini and Hochberg multiple testing correction adjusted p-value. For the i^th^ term (ranked according to p-value), the FDR q-value is the p-value multiplied by the number of GO terms assessed and divided by i.

### Statistical Analysis

All the statistical analyses were performed with either parametric or non-parametric tests depending on the normality of each compared data set, as determined by the D’Agostino & Pearson test using Prism 6.0 software (GraphPad, La Jolla, CA, USA). For multiple comparisons, either the non-parametric Friedman test with Dunn’s correction or the one-way ANOVA test with Geisser–Greenhouse correction were used, and analogously, either paired t tests or Wilcoxon tests for the comparisons between two groups if they were normally distributed or not, respectively. Results were expressed as mean ± SD, unless noted otherwise, and they were considered statistically significant when p < 0.05.

## Results

### Functional and Phenotypical Characteristics of TT-Loaded vitD3-tolDC

Monocytes from 16 healthy donor samples were isolated (94.4 ± 2.8% purity) with viabilities of CD14^+^ cells above 95%. After their differentiation into DC, with or without exposition to TT, cells were harvested and their purity, viability, and phenotype were determined by flow cytometry, as shown previously (25). In all cases, purity was >90%, as determined by the percentage of CD11c^+^ cells, with a mean viability of 94.2 ± 3.3%, which was not affected by the addition of TT (**Supplementary Table 2**). The study of the phenotype of vitD3-tolDC-TT showed significant reductions in the surface expression of CD86 (77.2 ± 8.7%) and HLA-DR (79.5 ± 7.7%) compared to mDC, but more importantly, evidenced that the exposure of DC to TT on day 3 of the culture did not have an effect *per se* over the expression of these molecules, neither in vitD3-tolDC nor in mDC, since there were no relevant differences on the percentages of reduction (**Supplementary Figure 1**). The same could be observed regarding the functionality of these cells. On the one hand, as also shown in **Supplementary Figure 1**, both vitD3-tolDC and vitD3-tolDC-TT exhibited a similar and strongly reduced induction of allogeneic proliferation compared to mDC (vitD3-tolDC: 50.6 ± 30.7, p < 0.001; vitD3-tolDC-TT: 49.2 ± 36.7, p = 0.001). On the other hand, there were no statistically significant differences in the mean induction of allogeneic proliferation induced by mDC-TT compared to mDC (p = 0.916). Altogether, our results evidence that vitD3-tolDC-TT show the same tolerogenic properties as vitD3-tolDC, thus demonstrating that loading these cells with TT does not affect their phenotype nor their functionality.

### VitD3-tolDC-TT Induce an Antigen-Specific Response Over Autologous PBMC

**Figure 1:**
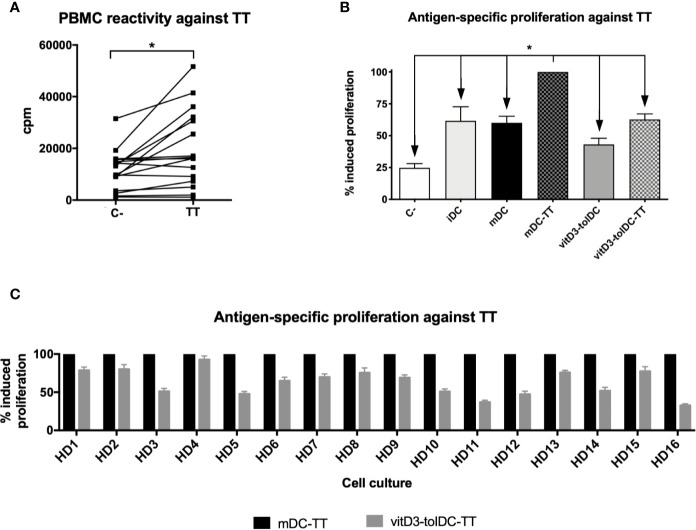
PBMC reactivity and antigen-specific induction of autologous proliferation mediated by DC against tetanus toxin. **(A)** Induction of proliferation of PBMC without stimuli (C−) and against tetanus toxin (TT) after 5 days of culture (n = 16). Data presented as counts per minute (cpm), measured as tritiated thymidine incorporation after 18 h. Ten replicated measurements of each condition were performed. **(B)** Induction of antigen-specific autologous proliferation against TT mediated by immature DC (iDC), mature DC (mDC), TT-loaded mDC (mDC-TT), vitamin D3-induced tolerogenic DC (vitD3-tolDC) and TT-loaded vitD3-tolDC (vitD3-tolDC-TT), as well as a negative control (C−), without any stimuli (n = 16) and **(C)** comparison of autologous antigen-specific proliferation against TT mediated by mDC-TT and vitD3-tolDC-TT on each donor. Data presented as relative percentage of induced proliferation compared to mDC-TT, measured as tritiated thymidine incorporation after 18 h. Six replicated measurements of each condition were performed. Error bars corresponding to SEM. ns, not significant; *p < 0.05. One-way ANOVA test with Geisser**–**Greenhouse correction or paired t test.

In order to test the antigen-specific functionality of vitD3-tolDC-TT in an autologous setup, we assessed the baseline reactivity of each donor against the TT itself to measure their potential to respond under these conditions. As shown in **Figure 1A**, we were able to assess the TT reactivity in all of our healthy donors, but only nine of them resulted positive, according to the criteria described in the *Material and Methods* section—when the mean proliferation of at least 5 out of 10 replicates was over the mean plus two times the SD of the control condition—and reaching statistical significance (p < 0.05).

Subsequently, we analyzed the capability of our cells to induce proliferation over autologous PBMC. As shown in **Figure 1B**, a significant proliferation was only induced by mDC-TT, as evidenced by the statistically significant differences observed with the remaining conditions. Specifically, reductions of a 38.4 ± 44.3% (p = 0.020), a 40.0 ± 21.0% (p < 0.001), a 56.9 ± 19.2% (p < 0.001) and a 37.3 ± 17.4% (p < 0.001) were observed in iDC, mDC, vitD3-tolDC and vitD3-tolDC-TT, respectively, compared to mDC-TT. Our results therefore evidence that autologous proliferation is only primed if an antigenic peptide is presented by an immunogenic DC condition, such as mDC-TT, confirming the antigen-specific modulation developed by our cells. Furthermore, reduced autologous proliferation mediated by vitD3-tolDC-TT was observed in all donors (**Figure 1C**).

### VitD3-tolDC-TT Drive a Reduction of T_H_1 CD4^+^ Cell Subpopulations

**Figure 2:**
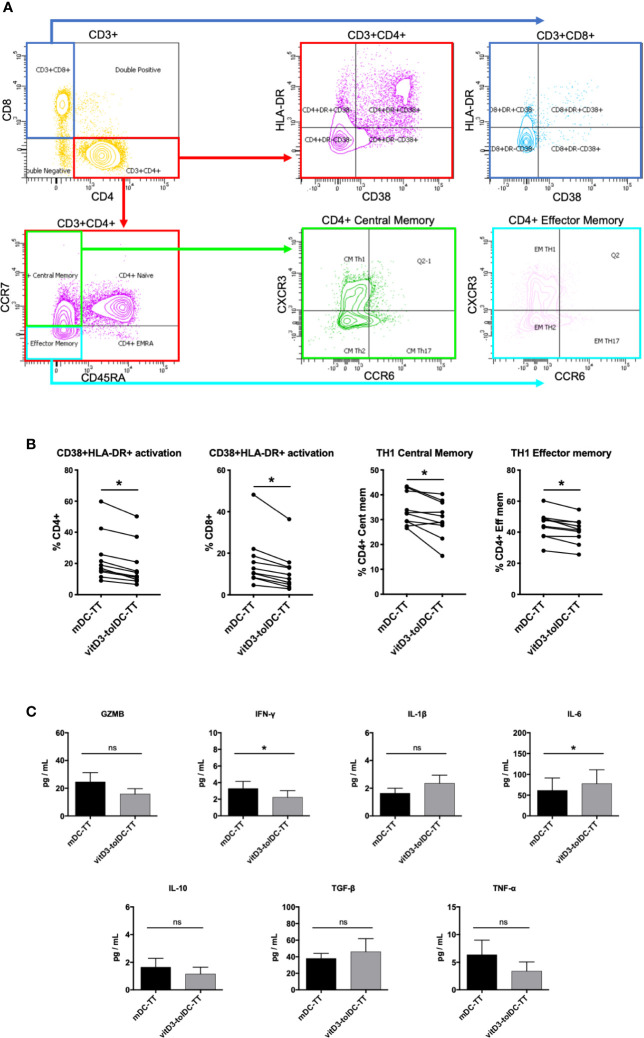
Phenotype and cytokine production of DC-co-cultured autologous PBMC. **(A)** Gating strategy. **(B)** Relative percentages of CD4^+^CD38^+^HLA-DR^+^, CD8^+^CD38^+^HLA-DR^+^, T_H_1 Central Memory and T_H_1 Effector Memory subpopulations on PBMC co-cultured with either tetanus toxin (TT)-loaded mature DC (mDC-TT) or TT-loaded vitamin D3-induced tolerogenic DC (vitD3-tolDC-TT) after 5 days of culture (n = 10). Data presented as the relative percentage of each lymphocyte subpopulation within its respective parent subpopulation, measured by multiparametric flow cytometry, in a box and whiskers representation. Error bars corresponding to the maximum and minimum values of each condition. **(C)** Analysis of the secretion of granzyme B (GZMB) (n = 12), IFN-*γ* (n = 12), IL-1β (n = 8), IL-6 (n = 8), IL-10 (n = 10), TGF-*β* (n = 7), and TNF-α (n = 8) in the supernatants of PBMC co-cultured with either mDC-TT or vitD3-tolDC-TT after 5 days of culture using either ELISA (TGF-*β*) or cytometric bead array (GZMB, IFN-*γ*, IL-1β, IL-6, IL-10, and TNF-α) techniques. One ELISA experiment was performed for all samples, with duplicated measurements for each sample. One single cytometric bead array experiment was performed for the analysis of all the samples, with one single measurement for each sample. Sample sizes vary due to some measurements being negative or under the detection limit of the technique. Error bars corresponding to SEM. ns, not significant; * p < 0.05. Paired t test or Wilcoxon test.

Once determined that an antigen-specific modulation was established by TT-loaded DC, we studied which changes were being induced over the autologous T lymphocytes. Therefore, we characterized the phenotype of CD3^+^CD4^+^ and CD3^+^CD8^+^ cells using an exhaustive multiparametric flow cytometry panel, described in previous studies (29). First, our results evidenced a reduction in the prevalence of activated T CD4^+^ cells, determined by HLA-DR and/or CD38 staining, in vitD3-Tcell compared to mDC-Tcell (Activated CD4^+^_mDC-Tcell_: 23.57 ± 15.81 *vs* Activated CD4^+^_vitD3-Tcell_: 18.52 ± 14.16; p = 0.002). The same effect was observed over T CD8^+^ cells (Activated CD8^+^_mDC-Tcell_: 15.94 ± 12.48 *vs* Activated CD8^+^_vitD3-Tcell_: 11.33 ± 9.81; p = 0.002). Furthermore, we found a reduction in the relative percentages of CD4^+^ T_H_1 Central Memory (CM) and Effector Memory (EM) subpopulations in vitD3-T cell (T_H_1 CM _mDC-Tcell_: 33.98 ± 6.44 *vs* T_H_1 CM _vitD3-Tcell_: 30.23 ± 7.48; p = 0.013; T_H_1 EM _mDC-Tcell_: 44.46 ± 8.72 *vs* T_H_1 EM _vitD3-Tcell_: 40.95 ± 8.08; p = 0.001). All these results are shown in **Figures 2A, B**. Thus, our data suggest that vitD3-tolDC-TT are driving an antigen-specific switch towards a more anti-inflammatory—or less T_H_1-like—profile over T CD4^+^ lymphocytes. We could not detect any significant changes over any other T cell subpopulation, nor Treg nor Tr1 subpopulations (data not shown).

Next, we analyzed the cytokine secretion profile present in the autologous co-cultures of mDC-TT and vitD3-tolDC-TT. Our results, as shown in **Figure 2C**, evidenced a statistically significant increased secretion of the cytokine IL-6 and lower levels of IFN-*γ* in the co-culture of autologous PBMC with vitD3-tolDC-TT compared to mDC-TT (IL-6 _mDC-TT_: 61.4 ± 84.3 pg/ml *vs* IL-6 _vitD3-tolDC-TT_: 77.7 ± 94.5 pg/ml; p = 0.039; and IFN-*γ*_mDC-TT_: 3.3 ± 2.9 pg/ml *vs* IFN-γ _vitD3-tolDC-TT_: 2.2 ± 2.8 pg/ml; p = 0.002). Therefore, the reduction in the production of IFN-*γ*, combined with the increase of IL-6, again suggest a reduction of the T_H_1-like cytokine profile, in line with the phenotype results. No statistically significant changes could be found in the production of GZMB, IL-1β, IL-10, TGF-β nor TNF-α.

### VitD3-tolDC Induce a General Transcriptomic Repression Over T CD4^+^ Cells

For all the 16 donors, at least 700,000 CD3^+^CD4^+^ cells in both conditions (mDC-Tcell and vitD3-Tcell) were successfully isolated by flow cytometry cell sorting. The gating strategy is shown in **Supplementary Figure 1**. Afterwards, we extracted their RNA and selected 10 donors that showed sufficient nucleic acid concentration and integrity for the RNA-seq analysis (RIN > 7) in both mDC-Tcell and vitD3-Tcell conditions. Consequently, donors HD4, HD5, HD9, HD10, HD11, and HD12 were discarded from downstream studies.

**Figure 3:**
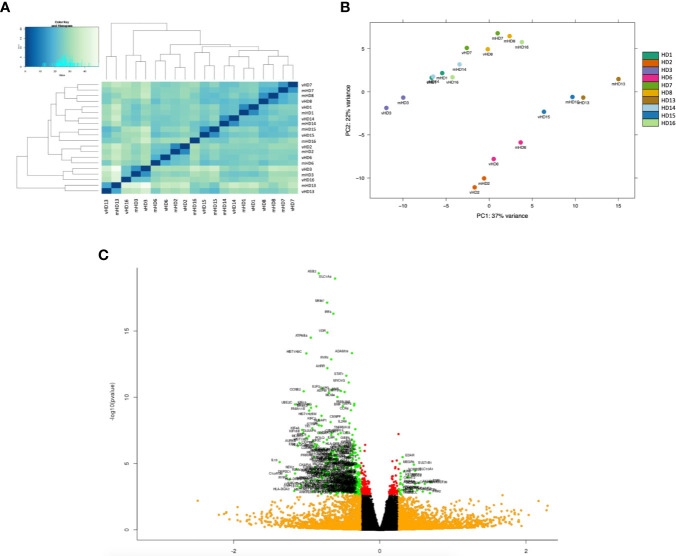
Exploratory analysis of the RNA-seq study of T CD4+ cells co-cultured with autologous antigen-specific DC. **(A)** Hierarchical clustering analysis by gene expression of the 20 samples of CD4^+^ T cells of the RNA-seq study. **(B)** Representation of the first two principal component analysis (PCA) components at the gene level. Each color corresponds to a different sample of CD4+ T cells, as depicted in the legend, and the co-culture condition of each sample is indicated with a prefix, either “m” for tetanus toxin (TT)-loaded mature DC or “v” for TT-loaded vitamin D3-induced tolerogenic DC. **(C)** Volcano plot showing the significant differentially expressed genes. Axis is the log2 fold change. Color code: green, significantly regulated genes (padj < 0.05; |FC| > 1.2) considered in the Gene Ontology enrichment analysis; orange, genes with |FC| > 1.2 below the significance threshold; red, genes with padj < 0.05 below the relevant fold change cutoff.

After processing the samples through the RNA-seq analysis, 39% of total reads could be assigned to different known RNA classes (**Supplementary Figure 2A**), and out of them, around 47% of these assigned reads could be related to protein coding genes (**Supplementary Figure 2B**). Interestingly, the hierarchical clustering analysis revealed that our samples tended to cluster by individual rather than by treatment (**Figure 3A**), but also that there is a consistent pattern by which vitD3-Tcell samples ranked higher on both axes from the PCA (**Figure 3B**). These results led to choose a paired comparative analysis approach for the differential expression analysis.

After the subsequent filtering process described in the *Material and Methods* section, a total of 16,333 protein coding genes with detectable reads were tested for differential expression. Among all of them, 546 genes showed a statistically significant change in their expression (adjusted p < 0.05) in vitD3-Tcell compared to mDC-Tcell, and only 373 also presented an absolute value of FC superior to 1.20 (|FC _vitD3-Tcell_ *_vs_*_mDC-Tcell_| > 1.20). While only 29 of these genes were up-modulated in vitD3-Tcell compared to mDC-Tcell, the majority of them, 344 genes, were down-modulated, indicating a strong transcriptomic repression induced by vitD3-tolDC-TT over these cells (**Figure 3C**).

### T CD4^+^ Cells Selectively Undergo a Strong Functional and Immune-Related Transcriptomic Down-Modulation Upon Interaction With vitD3-tolDC

When we studied those differentially expressed genes that appeared up-modulated (FC _vitD3-Tcell_ *_vs_*_mDC-Tcell_ > 1.20) in our analysis (**Table 1**), we did not find many relevant or immune-related genes. Specifically, 18 of these 29 genes did not have any GO annotation, and among the rest, we could only find the genes encoding the JUNB and SCML1 transcription factors and several other genes encoding different molecule transporters (*ABCC2* and *SLC10A1*), G-protein modulators (*GRTP1* and *RASA4*) and kinases (*AK5* and *CKMT2*).

**Table 1:** Up-modulated genes in vitD3-Tcell compared to mDC–Tcell.

Gene Symbol	GO annotation	FC vs mDC–Tcell	Adj. p-value
*TMIE*	NA	1.62	0.01199
*PRH2*	NA	1.61	0.04136
*ARHGEF26*	NA	1.53	0.01404
*GRTP1*	G-Protein Modulator; Cysteine Protease	1.50	0.03547
*AKAP6*	NA	1.45	0.01310
*CKMT2*	Amino Acid Kinase	1.43	0.03068
*SLC10A1*	Cation Transporter	1.40	0.00264
*C17orf107*	NA	1.40	0.01419
*SULT1B1*	NA	1.38	0.00117
*TTC16*	NA	1.37	0.03271
*TEC*	NA	1.35	0.02676
*KRT72*	NA	1.31	0.00543
*ABCC2*	ATP-Binding Cassette (ABC) Transporter	1.30	0.00598
*KRT73*	NA	1.28	0.02747
*SORBS3*	NA	1.26	0.00279
*AK5*	Nucleotide Kinase	1.24	0.02996
*EDAR*	NA	1.24	0.00041
*ADAM23*	Metalloprotease	1.24	0.01495
*RALGPS2*	Guanyl-Nucleotide Exchange Factor	1.23	0.02922
*ALS2CL*	NA	1.22	0.04815
*KBTBD11*	NA	1.22	0.00543
*JUNB*	Basic Leucine Zipper Transcription Factor; Nucleic Acid Binding	1.22	0.00360
*RASA4*	G-Protein Modulator	1.22	0.01328
*C9orf72*	NA	1.22	0.00818
*ZC4H2*	NA	1.21	0.04572
*ADPRM*	NA	1.21	0.01438
*SCML1*	Chromatin/Chromatin-Binding Protein; Transcription Factor	1.21	0.03955
*MEGF6*	Extracellular Matrix Protein	1.21	0.00102
*LMTK3*	NA	1.21	0.03156

Gene expression values from an RNA-seq analysis with healthy donors (n = 10). Data presented as the mean fold change (FC) of expression in vitD3-Tcell compared to mDC-Tcell. GO, Gene Ontology; mDC-Tcell, mature dendritic cell-conditioned CD3^+^CD4^+^ cells; NA, not available; padj, adjusted p-value; vitD3-Tcell, vitamin D3-induced tolerogenic dendritic cell-conditioned CD3^+^CD4^+^ cells.

However, as mentioned above, the study of the down-modulated genes (FC _vitD3-Tcell_ *_vs_*_mDC-Tcell_ < 1.20) yielded many more results. Within the 50 most down-regulated results (FC _vitD3-Tcell_ *_vs_*_mDC-Tcell_ < 1.78) we found several genes encoding proteins involved in the immune response (*CCL17*, *CCL22*, *EBI3*, *IL13* and *LIF*), antigen presentation (*HLA-DQA1*, *HLA-DQA2* and *HLA-DRB5*) and microtubule binding (*KIF4A*, *KIF15*, *KIF18B* and *KIFC1*), among others (**Table 2**). Furthermore, when we analyzed the whole list, we could find not only several more genes included in these categories, but also many other genes encoding proteins related to cytoskeleton and cell adhesion (*ARPC1B*, *CAPG*, *CTNNA1*, *LGALS1*, *MYL6B*, *LGALS9* or *SDC4*), actin related functionalities (*ACTB*, *ACTG1*, *PARVB* or *TPM4*), G-proteins and modulators (*GBP2*, *GBP4*, *GNA15*, *GNG4*, *IQGAP3*, *MYO1G*, *MYO1E* or *SRGAP3*), nucleic acid binding (*ASF1B*, *DEPDC1*, *EXO1* or *FEN1*), histones (*HIST1H2BL*, *HIST2H2BF* or, *HIST1H4H*), the pro-inflammatory transcription factor STAT1 and other pro-inflammatory mediators (*TNFSF4*), different kinase activators and modulators, proteases and protease inhibitors, oxydases, oxygenases, transferases and many other metabolic mediators. The whole list is shown in **Supplementary Table 3**. Altogether, these results indicate that vitD3-tolDC-TT mediate a strong down-modulation of metabolic and immune-related functions over vitD3-Tcell.

**Table 2:** Top 50 down-modulated genes in vitD3-Tcell compared to mDC-Tcell.

GeneSymbol	GO annotation	FC vs mDC-Tcell	padj
*IL13*	Cytokine	−2.59	0.00082
*C1orf106*	NA	−2.43	0.00486
*RYR2*	Ligand-Gated Ion Channel	−2.35	0.00802
*HLA-DQA2*	Major Histocompatibility Complex Antigen	−2.25	0.03271
*UBE2C*	NA	−2.23	0.00000
*DEPDC1*	Nucleic Acid Binding	−2.23	0.00367
*NEK2*	Protein Kinase	−2.20	0.00206
*EBI3*	Cytokine; Defense/Immunity Protein	−2.18	0.00009
*CCL17*	Chemokine	−2.18	0.01852
*AURKB*	Non-Receptor Serine/Threonine Protein Kinase	−2.14	0.00006
*CYP1B1*	Oxygenase	−2.12	0.02334
*SPC25*	Enzyme Modulator	−2.11	0.01556
*KIF4A*	Microtubule Binding Motor Protein	−2.09	0.00001
*KIF18B*	Microtubule Binding Motor Protein	−2.07	0.00001
*CCNB2*	Kinase Activator	−2.06	0.00000
*HLA-DRB5*	Major Histocompatibility Complex Antigen	−2.02	0.00970
*MCM10*	NA	−2.01	0.00003
*HIST1H3C*	Histone	−2.01	0.00000
*KIF15*	Microtubule Binding Motor Protein	−1.97	0.00000
*BIRC5*	Protease Inhibitor	−1.96	0.00002
*CHST3*	NA	−1.95	0.01782
*FAM111B*	NA	−1.95	0.00000
*MYBL2*	NA	−1.92	0.00000
*ATP8B4*	Cation Transporter. Hydrolase	−1.92	0.00000
*SKA1*	NA	−1.91	0.00898
*KIAA0101*	NA	−1.91	0.00292
*TK1*	Nucleotide Kinase	−1.90	0.00001
*HIST1H3J*	Nucleic Acid Binding; Transcription Factor	−1.89	0.03504
*E2F8*	Nucleic Acid Binding; Transcription Factor	−1.89	0.00209
*HLA-DQA1*	Major Histocompatibility Complex Antigen	−1.88	0.00011
*HIST1H3F*	Reductase	−1.88	0.00005
*RRM2*	Reductase	−1.88	0.00337
*GNG4*	Heterotrimeric G-Protein	−1.87	0.01479
*PRR11*	NA	−1.86	0.00041
*CEP55*	NA	−1.85	0.00008
*CKAP2L*	NA	−1.84	0.00151
*CDCA8*	NA	−1.84	0.00015
*HIST1H3G*	Histone	−1.83	0.00000
*CDK1*	Non-Receptor Serine/Threonine Protein Kinase; Non-Receptor Tyrosine Protein Kinase	−1.83	0.00283
*HMMR*	NA	−1.83	0.00012
*PKMYT1*	Non-Receptor Serine/Threonine Protein Kinase	−1.83	0.00825
*CCL22*	Chemokine	−1.82	0.02508
*CREB3L3*	NA	−1.81	0.02749
*CDC25A*	Protein Phosphatase	−1.81	0.02454
*DTL*	NA	−1.81	0.00008
*RAD51AP1*	NA	−1.80	0.00912
*ESCO2*	NA	−1.79	0.01020
*LIF*	Cytokine	−1.78	0.00000
*KIFC1*	Microtubule Binding Motor Protein	−1.78	0.00000
*ASB2*	NA	−1.78	0.00000

Gene expression values from an RNA-seq analysis with healthy donors (n = 10). Data presented as the mean fold change (FC) of expression in vitD3-Tcell compared to mDC-Tcell. GO, Gene Ontology; mDC-Tcell, mature dendritic cell-conditioned CD3^+^CD4^+^ cells; NA, not available; padj, adjusted p-value; vitD3-Tcell, vitamin D3-induced tolerogenic dendritic cell-conditioned CD3^+^CD4^+^ cells.

### VitD3-Tcell Present Decreased Cell Cycle and Mitotic Activity

In this regard, the GO enrichment analysis further supported the results observed in the differential gene expression (DGE) study. Thus, first, the enrichment analysis produced a total of 482 protein sets and pathways with p < 0.001, four of them up-modulated—although none of them showed an FDR value below 0.25—and the remaining 478 down-modulated (**Supplementary Table 4**). We further filtered the results to analyze only those GO terms that presented a much more significant enrichment (p < 10^-9^). This process left us with a total of 66 down-modulated GO terms, but none up-modulated. These 66 elements, ordered by decreasing ES, are shown in **Table 3**. Interestingly, among the most significantly enriched down-modulated pathways, we found several GO annotations referring to immune-related functionality (for instance *Interferon-Gamma-Mediated Signaling Pathway*, ES: 10.65; *Cytokine-Mediated Signaling Pathway*, ES: 5.09; or *Immune Response*, ES: 3.03), class II-related antigen presentation (like *MHC Class II Protein Complex*, ES: 30.23; *Antigen Processing And Presentation Of Exogenous Peptide Antigen Via MHC Class II*, ES: 8.08; or *Antigen Processing And Presentation Of Exogenous Peptide Antigen*, ES: 6.74), cell response to different stimuli (*Cell Surface Receptor Signaling Pathway*, ES: 2.21; *Response To Stress*, ES: 1.93; or *Cellular Response To Stimulus*, ES: 1.80) and, specially, to cell cycle and mitotic division (for instance *Condensed Chromosome Outer Kinetochore*, ES: 21.98; *Mitotic Spindle Organization*, ES: 8.79; *Microtubule Cytoskeleton Organization Involved In Mitosis*, ES: 7.48; or *Cell Cycle Checkpoint*, ES: 6.05). Our results, in line with the DGE analysis, would suggest that vitD3-Tcell are undergoing a process of transcriptomic down-modulation leading to reduced immune-related, metabolic and proliferative functionalities.

**Table 3:** Most significantly down-regulated Gene Ontology terms in vitD3-Tcell compared to mDC-Tcell.

GO category	GO term	ES	p-value	FDR
GOCOMPONENT	MHC Class II Protein Complex	30.23	5.24E-16	2.39E-13
GOCOMPONENT	Condensed Chromosome Outer Kinetochore	21.98	3.96E-10	4.81E-08
GOCOMPONENT	MHC Protein Complex	18.71	1.94E-12	5.88E-10
GOCOMPONENT	Clathrin-Coated Endocytic Vesicle Membrane	16.92	6.86E-10	7.81E-08
GOFUNCTION	Peptide Antigen Binding	16.92	6.86E-10	7.06E-07
GOPROCESS	Interferon-Gamma-Mediated Signaling Pathway	10.65	1.35E-13	1.44E-10
GOCOMPONENT	DNA Packaging Complex	10.26	5.61E-19	5.11E-16
GOPROCESS	Nuclear Chromosome Segregation	10.21	9.93E-10	2.98E-07
GOCOMPONENT	Nucleosome	9.82	2.16E-16	1.31E-13
GOPROCESS	Mitotic Spindle Organization	8.79	1.87E-11	8.07E-09
GOPROCESS	Antigen Processing And Presentation Of Exogenous Peptide Antigen Via MHC Class II	8.08	1.20E-12	8.70E-10
GOPROCESS	Regulation Of Chromosome Segregation	7.98	9.85E-14	1.24E-10
GOPROCESS	Antigen Processing And Presentation Of Peptide Or Polysaccharide Antigen Via MHC Class II	7.98	1.50E-12	9.88E-10
GOPROCESS	Antigen Processing And Presentation Of Peptide Antigen Via MHC Class II	7.98	1.50E-12	9.43E-10
GOPROCESS	Microtubule Cytoskeleton Organization Involved In Mitosis	7.48	1.93E-11	8.07E-09
GOPROCESS	Chromosome Segregation	7.21	8.19E-13	7.07E-10
GOPROCESS	Mitotic Cell Cycle	7.20	1.49E-15	2.29E-12
GOPROCESS	Nucleosome Assembly	7.08	9.78E-14	1.35E-10
GOPROCESS	Antigen Processing And Presentation Of Exogenous Peptide Antigen	6.74	1.09E-11	5.59E-09
GOPROCESS	Antigen Processing And Presentation Of Exogenous Antigen	6.62	1.57E-11	6.98E-09
GOCOMPONENT	Protein-DNA Complex	6.57	5.01E-14	1.82E-11
GOPROCESS	Antigen Processing And Presentation Of Peptide Antigen	6.32	3.73E-11	1.47E-08
GOPROCESS	Spindle Organization	6.11	2.12E-10	7.32E-08
GOPROCESS	Cell Cycle Checkpoint	6.05	2.87E-11	1.16E-08
GOPROCESS	Antigen Processing And Presentation	5.91	1.55E-11	7.15E-09
GOCOMPONENT	Midbody	5.75	9.66E-12	1.95E-09
GOPROCESS	Nucleosome Organization	5.71	1.12E-11	5.52E-09
GOPROCESS	Regulation Of Mitotic Nuclear Division	5.46	7.83E-11	2.92E-08
GOPROCESS	Regulation Of Nuclear Division	5.39	1.37E-11	6.52E-09
GOPROCESS	Cytokine-Mediated Signaling Pathway	5.09	3.61E-27	2.49E-23
GOCOMPONENT	Spindle	5.07	1.32E-10	1.85E-08
GOCOMPONENT	Chromosome	4.67	9.10E-12	2.07E-09
GOPROCESS	Mitotic Cell Cycle Process	4.64	3.48E-28	4.81E-24
GOPROCESS	Positive Regulation Of Cell Cycle Process	4.55	4.10E-11	1.57E-08
GOPROCESS	Protein-DNA Complex Assembly	4.47	7.59E-10	2.33E-07
GOPROCESS	Cell Division	4.25	5.31E-16	9.17E-13
GOPROCESS	Negative Regulation Of Cell Cycle Process	4.04	6.43E-10	2.02E-07
GOPROCESS	Regulation Of Mitotic Cell Cycle Phase Transition	3.99	1.04E-12	8.42E-10
GOPROCESS	Chromosome Organization	3.94	1.50E-12	1.04E-09
GOPROCESS	Regulation Of Cell Cycle Phase Transition	3.82	1.92E-12	1.15E-09
GOPROCESS	Cell Cycle Process	3.63	2.27E-25	1.05E-21
GOPROCESS	Cell Cycle	3.56	1.11E-12	8.53E-10
GOPROCESS	Regulation Of Cell Cycle Process	3.47	7.37E-17	1.69E-13
GOFUNCTION	Protein Heterodimerization Activity	3.44	5.36E-10	7.36E-07
GOCOMPONENT	Chromosomal Part	3.27	8.53E-20	1.55E-16
GOCOMPONENT	Nuclear Chromosome Part	3.14	3.70E-11	6.75E-09
GOPROCESS	Regulation Of Mitotic Cell Cycle	3.12	2.72E-12	1.44E-09
GOPROCESS	Negative Regulation Of Cell Cycle	3.12	2.28E-10	7.66E-08
GOPROCESS	Immune Response	3.03	2.51E-12	1.45E-09
GOPROCESS	Regulation Of Cell Cycle	2.82	6.87E-17	1.90E-13
GOPROCESS	Positive Regulation Of Immune System Process	2.68	5.07E-10	1.67E-07
GOFUNCTION	Protein Dimerization Activity	2.58	1.83E-12	3.76E-09
GOPROCESS	Immune System Process	2.36	3.18E-16	6.27E-13
GOPROCESS	Regulation Of Immune System Process	2.31	1.35E-10	4.91E-08
GOCOMPONENT	Cytoskeletal Part	2.26	7.15E-12	1.86E-09
GOPROCESS	Cell Surface Receptor Signaling Pathway	2.21	2.80E-13	2.76E-10
GOPROCESS	Response To Stress	1.93	6.04E-13	5.56E-10
GOCOMPONENT	Extracellular Region Part	1.83	1.79E-10	2.33E-08
GOCOMPONENT	Non-Membrane-Bounded Organelle	1.82	1.01E-10	1.67E-08
GOCOMPONENT	Intracellular Non-Membrane-Bounded Organelle	1.82	1.01E-10	1.53E-08
GOPROCESS	Cellular Response To Stimulus	1.80	6.19E-10	1.99E-07
GOPROCESS	Response To Stimulus	1.78	6.82E-17	2.35E-13
GOPROCESS	Signal Transduction	1.76	9.95E-14	1.14E-10
GOPROCESS	Regulation Of Cellular Process	1.28	1.91E-10	6.76E-08
GOFUNCTION	Protein Binding	1.26	4.87E-15	2.01E-11
GOPROCESS	Cellular Process	1.15	2.64E-12	1.46E-09

Results presented as Enrichment Score (ES) values of different Gene Ontology (GO) terms in vitD3-Tcell compared to mDC-Tcell using data from an RNA-seq analysis with healthy donors (n = 10). FDR, False Discovery Rate; GOCOMPONENT, GO cellular component; GOFUNCTION, GO molecular function; GOPROCESS, GO biological process; mDC-Tcell, mature dendritic cell-conditioned CD3^+^CD4^+^ cells; NA, not available; padj, adjusted p-value; vitD3-Tcell, vitamin D3-induced tolerogenic dendritic cell-conditioned CD3^+^CD4^+^ cells.

## Discussion

In this study we analyzed the specific effect of vitD3-tolDC over autologous CD4^+^ T cells. Thus, we switched the attention from the study of tolDC themselves—widely studied so far—to focus on the study of the functional effect that these cells develop over T cells upon their interaction. In homeostatic conditions, either depletion, inactivation and/or induction of anergy is often induced on T cells due to a lack of one or more of the three immunogenic activation signals. This causes T cells to become hyporesponsive or to die (20, 30). However, in the case of autoimmunity, where T cells are already activated and developing an immunogenic response, an antigen-specific process of tolerance induction is required. In this regard, previous *in vivo* studies with vitD3-tolDC in the EAE model showed that an antigen-specific setup—and therefore an active process—is required, provided that a beneficial effect of this therapy was only observed when vitD3-tolDC were pulsed with the adequate immunogenic peptide (23, 24). Consequently, we developed an experimental model for the generation of autologous antigen-specific vitD3-tolDC and T cells from healthy donors, using an immunogenic peptide presented *via* class II MHC with the aim to reproduce antigen presentation in the context of CD4^+^-mediated autoimmune diseases. In this regard, we selected TT for its compliance with this feature—since the vaccination against TT is included in European health systems—which also allowed us to use healthy donors instead of patients of a determined autoimmune disease, thus eliminating disease-conditioned immune variations.

All in all, our approach aimed to be as versatile as possible, and to serve as a preliminary study for future research, with the idea that the immunogenic peptide/s might be replaced depending on the disease of interest, as well as, of course, using patient cells instead of healthy donors. On the one hand, for autoimmune diseases with identified autoantigens, this decision would be trivial. On the other hand, for those conditions in which autoimmune antigens are yet to be identified, the experimental design should be considered case by case. For instance, using autologous synovial fluid as a source for autoantigens to load tolDC has provided promising results in rheumatoid arthritis (31), so analog workaround solutions could be taken into account.

After validating our experimental setup—meaning that vitD3-tolDC-TT were able to induce an antigen-specific response—we focused on the study of the actual phenotypic, functional and transcriptomic modulations induced by vitD3-tolDC. First, the analysis of the phenotype of T CD4^+^ cells evidenced that their interaction with vitD3-tolDC-TT caused a relative reduction in the activation of these cells. More importantly, a switch in the immune response of these cells towards a more immunoregulatory profile was induced, with a reduction in the prevalence of T_H_1 memory subpopulations. These results were further supported by the decrease of IFN-*γ* production in the autologous co-culture supernatants, consequently supporting that vitD3-tolDC were inducing a switch towards a more anti-inflammatory immune profile, in line with previous findings in the literature (16–18).

When we deepened into the analysis of the vitD3-tolDC-mediated transcriptomic profile of T cells, we observed several genes and GO terms regulated in line with the abovementioned phenotypical and functional switch towards a less activated an more immunoregulatory profile; for instance, a down-modulation of *STAT1* gene and the interferon-gamma-mediated signaling pathway was observed, which on the other hand supported the robustness of our RNA-seq study. Beyond this, the results pointed towards a generalized down-modulation of the transcriptomic profile of vitD3-Tcell that could either respond to an induction of T CD4^+^ cell hyporesponsiveness or even to a process of clonal deletion. On the one hand, the down-modulation of genes and pathways involved in crucial cellular mechanisms—in particular those related to cell proliferation, mitosis, cell cycle and response to immune stimuli, some of them never reported before—could be explained by both of these processes. However, on the other hand, the lack of induction of cell death and apoptotic-related pathways makes clonal deletion very unlikely to be happening. Therefore, our results suggest that the antigen-specific interaction of vitD3-tolDC with autologous T CD4^+^ cells is mediating, in fact, an induction of hyporesponsiveness over these cells. Furthermore, previous studies from our group already pointed in this direction (16), a biological situation that, potentially, might lead to the abrogation of an autoimmune immunogenic response in patients. Moreover, our current results provide evidence that these modulations are taking place at the transcriptomic level in T CD4^+^ cells, indicating that the antigen-specific modulation induced by vitD3-tolDC is deeper than expected and, in consequence, probably also long-lasting.

Unfortunately, the lack of strongly up-regulated genes among the protein-coding RNA transcripts did not allow us to point towards many clear candidate biomarkers that might become indicators of the response of T cells upon their interaction with vitD3-tolDC to monitor patients in clinical trials. One of the most relevant exceptions came given by *JUNB* gene, encoding a member of the AP-1 family of transcription factors. In experimental models, this gene has been reported to be crucial in maintaining Treg suppressive function (32), although it is also apparently involved in the induction and maintenance of IL-23-related pathogenicity of T_H_17 cells (33, 34). However, neither of these functionalities seems to fit in our model based on our results, since neither T_H_17 nor Treg induction was evidenced. Consequently, it would be interesting to elucidate the specific role of JunB in our experimental setting, and to what extent small changes in its expression can actually be sufficient or not for the induction and maintenance of immune tolerance. Furthermore, if this hypothesis proves to be valid, either *JUNB* and/or other related genes might also constitute potential biomarkers of response to vitD3-tolDC treatment in the clinic. It is also worth noting that we may have overlooked other potential biomarkers that might be found among the non-protein-coding and alternate splicing RNA transcripts. Although this possibility, if true, would have a limited functional value in our experimental model, it could be addressed in future studies.

In addition, our results did not allow us to reach any conclusion regarding a potential induction of anergy, and, as discussed above, they also rule out any kind of Treg or Tr1 response. Even though our previous *in vivo* experiments with the murine EAE model pointed towards an induction of Treg mediated by vitD3-tolDC (23, 24), we have observed that, at least in this experimental setting, this is not the case with human cells. These results are in line with what previous studies from both our group and other authors have already reported (13, 16, 35), although there seems to be some controversy (36, 37). However, it is also worth mentioning that in these reports, Treg induction was only observed after two rounds of stimulation of T cells, which might explain why we have not detected it. This is definitely something to be taken into account, since Treg induction is, undoubtedly, one of the main mechanisms for the induction of immune tolerance of tolDC and other antigen presenting cell approaches (38, 39). Indeed, Treg themselves, when expanded *in vitro*, present a huge therapeutic potential as a cell therapy for autoimmune diseases in humans (40). Consequently, the transcriptomic study of vitD3-tolDC-induced Treg should probably be addressed separately in future studies, since two rounds of T cell stimulation might have masked some of the results that we have reported here.

Our current study presents some limitations. First, since we focused on the study of CD4^+^ T cells alone, we were naturally omitting the potential modulation that vitD3-tolDC might be mediating through other subpopulations, such as regulatory B cells or regulatory NK cells. Furthermore, the election of the timepoint for the RNA-seq analysis intrinsically establishes another limitation, which is the status of the transcriptomic profile at different timepoints of the co-culture. However, our selection came based on the phenotypical and functional results shown in the study, which evidence that, by day 5 of the co-culture, there is a significant and differential modulation mediated by vitD3-tolDC over T CD4^+^ cells. Consequently, even though it is true that other timepoints might provide valuable additional information, we think that our election provided the best compromise, and a full time-course characterization of the antigen-specific transcriptomic changes induced by vitD3-tolDC will be addressed in future studies. On the other hand, we cannot fully discard the presence of non-antigen-specific CD4^+^ cells by the time the cell sorting was performed. However, even with a residual amount of non-antigen-specific T cells, the obtained results were consistent not only within the different techniques, but also with the literature, supporting our findings.

In conclusion, our results evidence that vitD3-tolDC are inducing a strong antigen-specific transcriptomic down-modulation over autologous T CD4^+^ cells, with a reduced ability to respond to immune- and non-immune-related stimuli. Consequently, it constitutes one of the first attempts to understand the changes that T cells are undergoing at the transcriptomic level upon an antigen-specific interaction with a tolerogenic cell product, such as vitD3-tolDC. In that regard, we identified several specific genes and pathways selectively down-modulated, as well as the induction of *JUNB*, which might constitute a putative biomarker of the modulation mediated by vitD3-tolDC over CD4^+^ T cells and, consequently, a potential biomarker to monitor the effect of vitD3-tolDC after their administration to patients. Therefore, the results presented in this article allowed us to better understand the process of T cell hyporesponsiveness at the molecular level and, more importantly, to set the path for future studies to fully elucidate the specific processes that are taking place in one of the most important mechanisms that the promising tolDC-based therapies can trigger in order to restore tolerance in autoimmune diseases.

## Data Availability Statement

The datasets presented in this study can be found in online repositories. The names of the repository/repositories and accession number(s) can be found below: https://www.ncbi.nlm.nih.gov/geo/, GSE128816.

## Ethics Statement

This study was reviewed and approved by the Germans Trias i Pujol Hospital Ethical Committee. The participants provided their written informed consent to participate in this study.

## Author Contributions

EM-C, JN-B and MM conceived the experiments. JN-B and MM performed the cell cultures and the cell sortings, and prepared the samples for the RNA-seq analysis. JN-B and MM analyzed the results. EM-C, JN-B and MM interpreted the results. JN-B wrote the manuscript. AT-S, BQ-S, CR-T, EM-C, JN-B, and MM reviewed the manuscript. All authors contributed to the article and approved the submitted version.

## Funding

This work was partially supported by projects PI14/01175, PI16/01737 and PI17/01521, integrated in the *Plan Nacional de I+D+I* and co-supported by the ISCIII-*Subdirección General de Evaluación* and the *Fondo Europeo de Desarrollo Regional* (FEDER), and by grant 140191 from IWT-TBM (Belgium). JN-B was beneficiary of a FI-DGR PhD contract from AGAUR (*Agència de Gestió d’Ajuts Universitaris i de Recerca*), supported by the Government of Catalonia (02/2015-01/2018). The contracts of JN-B and MM have been partially supported by grant 140191 from IWT-TBM. The authors are members of a consolidated group recognized by AGAUR (2017 SGR 103), supported by the Government of Catalonia. This work has been supported by positive discussion through A FACTT network (COST Action BM1305: www.afactt.eu) and project 779316–ReSToRe (www.h2020restore.eu). COST and ReSToRe are supported by the EU Framework Programme Horizon 2020.

## Conflict of Interest

The authors declare that the research was conducted in the absence of any commercial or financial relationships that could be construed as a potential conflict of interest.

## Supplementary Material

Supplementary Figure 1 Phenotype and functionality of DC. **(A)** Gating strategy. **(B)** Surface expression of CD86 (FITC) and HLA-DR (V500) on immature DC (iDC), mature DC (mDC), tetanus toxin (TT)-loaded mDC (mDC-TT), vitamin D3-induced tolerogenic DC (vitD3-tolDC) and TT-loaded vitD3-tolDC (vitD3-tolDC-TT) (n = 8). Data presented as relative percentage of median fluorescence intensity (MFI) normalized *versus* mDC, measured by flow cytometry. In all cases, DC populations were CD11c^+^ **(C)** Allogeneic proliferation of PBMC from healthy donors co-cultured with either iDC, mDC, mDC-TT, vitD3-tolDC or vitD3-tolDC-TT (n = 14) in a DC : PBMC 1:20 ratio. Data presented as relative percentage of induced proliferation compared to mDC, measured as tritiated thymidine incorporation after 18 h. Six replicated measurements of each condition were performed. Error bars corresponding to SEM. ns = not significant; * p < 0.05. Friedman test with Dunn’s correction. **(D)** Gating strategy for the sorting of autologous T CD4^+^ cells.

Supplementary Figure 2 Assignment and functional classification of the total reads from the RNA-seq study of T CD4^+^ cells co-cultured with autologous antigen-specific DC. **(A)** Assignment of the total reads from the RNA-seq analysis to known RNA classes. **(B)** Classification of the assigned reads into known RNA functionalities.

Supplementary Table 3 Up and down-modulated genes in vitD3-Tcell compared to mDC-Tcell.

Supplementary Table 4 Differentially enriched Gene Ontology terms in vitD3-Tcell compared to mDC-Tcell.
