# Supplementary figures and images for: Vitamin D3-Induced Tolerogenic Dendritic Cells Modulate the Transcriptomic Profile of T CD4+ Cells Towards a Functional Hyporesponsiveness
Source: Front Immunol. 2021 Jan 20;11:599623. doi: 10.3389/fimmu.2020.599623 (PMC7856150; doi:10.3389/fimmu.2020.599623)

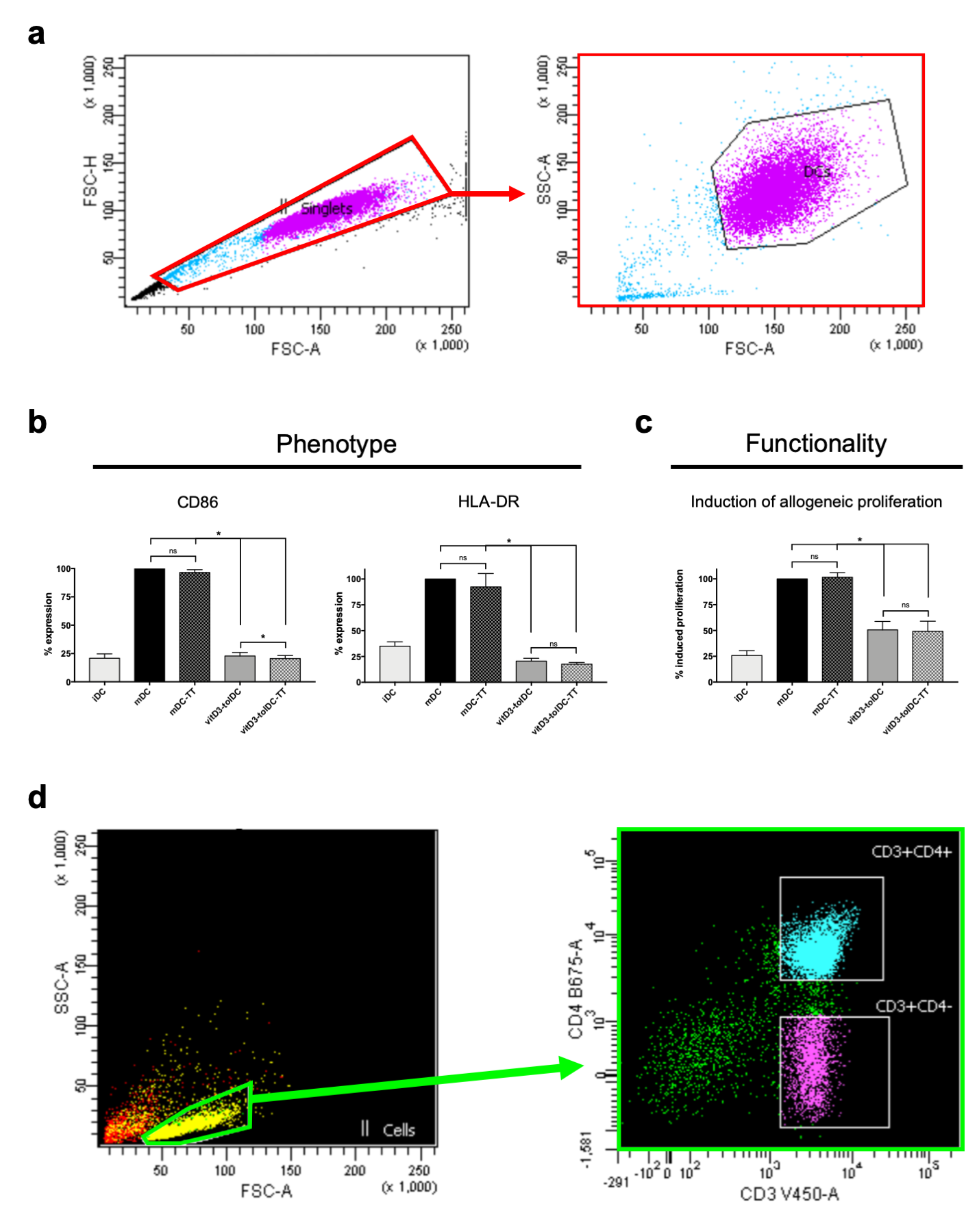

Supplement: Supplementary Figure 1 — Phenotype and functionality of DC. (A) Gating strategy. (B) Surface expression of CD86 (FITC) and HLA-DR (V500) on immature DC (iDC), mature DC (mDC), tetanus toxin (TT)-loaded mDC (mDC-TT), vitamin D3-induced tolerogenic DC (vitD3-tolDC) and TT-loaded vitD3-tolDC (vitD3-tolDC-TT) (n = 8). Data presented as relative percentage of median fluorescence intensity (MFI) normalized versus mDC, measured by flow cytometry. In all cases, DC populations were CD11c+ (C) Allogeneic proliferation of PBMC from healthy donors co-cultured with either iDC, mDC, mDC-TT, vitD3-tolDC or vitD3-tolDC-TT (n = 14) in a DC : PBMC 1:20 ratio. Data presented as relative percentage of induced proliferation compared to mDC, measured as tritiated thymidine incorporation after 18 h. Six replicated measurements of each condition were performed. Error bars corresponding to SEM. ns = not significant; * p < 0.05. Friedman test with Dunn’s correction. (D) Gating strategy for the sorting of autologous T CD4+ cells. [file Image_1.tiff]

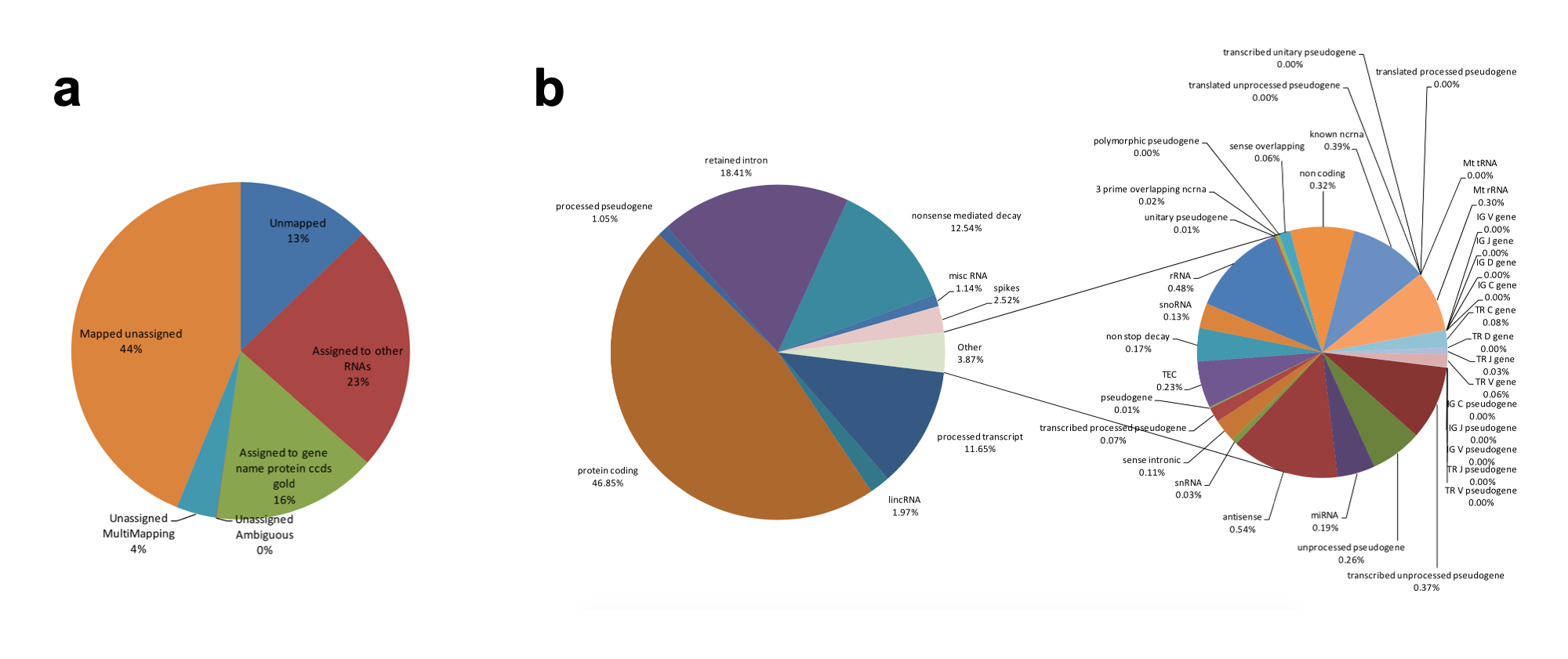

Supplement: Supplementary Figure 2 — Assignment and functional classification of the total reads from the RNA-seq study of T CD4+ cells co-cultured with autologous antigen-specific DC. (A) Assignment of the total reads from the RNA-seq analysis to known RNA classes. (B) Classification of the assigned reads into known RNA functionalities. [file Image_2.tiff]
