# Supplementary material for: Vitamin D3-Induced Tolerogenic Dendritic Cells Modulate the Transcriptomic Profile of T CD4+ Cells Towards a Functional Hyporesponsiveness
Source: Front Immunol. 2021 Jan 20;11:599623. doi: 10.3389/fimmu.2020.599623 (PMC7856150; doi:10.3389/fimmu.2020.599623)
Supplement: Supplementary file 3 [file Table_1.doc]

**Supplementary Table 1.** Definition of T cell subpopulations in the flow cytometry panel.

| **Cell population** | **Marker signature** |
| --- | --- |
| **T lymphocytes** | **CD3+** |
| **T CD4 lymphocytes** | CD3+**CD4+** |
| **CD4 Naïve** | CD3+CD4+**CCR7+CD45RA+** |
| **CD4 Central Memory** | CD3+CD4+**CCR7+CD45RA-** |
| **T_H_1** | CD3+CD4+CCR7+CD45RA-**CCR6-CXCR3+** |
| **T_H_2** | CD3+CD4+CCR7+CD45RA-**CCR6-CXCR3-** |
| **T_H_17** | CD3+CD4+CCR7+CD45RA-**CCR6+CXCR3-** |
| **T_H_1T_H_17** | CD3+CD4+CCR7+CD45RA-**CCR6+CXCR3+** |
| **CD4 Effector Memory** | CD3+CD4+**CCR7-CD45RA-** |
| **T_H_1** | CD3+CD4+CCR7-CD45RA-**CCR6-CXCR3+** |
| **T_H_2** | CD3+CD4+CCR7-CD45RA-**CCR6-CXCR3-** |
| **T_H_17** | CD3+CD4+CCR7-CD45RA-**CCR6+CXCR3-** |
| **T_H_1/17** | CD3+CD4+CCR7-CD45RA-**CCR6+CXCR3+** |
| **CD4 EMRA** | CD3+CD4+**CCR7-CD45RA+** |
| **Treg** | CD3+CD4+**CCR4+CD25+CD127low** |
| **Tr1** | CD3+CD4+**CD49b+LAG-3+** |
| **T CD8 lymphocytes** | CD3+**CD8+** |
| **CD8 Naïve** | CD3+CD8+**CCR7+CD45RA+** |
| **CD8 Central Memory** | CD3+CD8+**CCR7+CD45RA-** |
| **CD8 Effector Memory** | CD3+CD8+**CCR7-CD45RA-** |
| **CD8 TEMRA** | CD3+CD8+**CCR7-CD45RA+** |
