# Supplementary material for: Vitamin D3-Induced Tolerogenic Dendritic Cells Modulate the Transcriptomic Profile of T CD4+ Cells Towards a Functional Hyporesponsiveness
Source: Front Immunol. 2021 Jan 20;11:599623. doi: 10.3389/fimmu.2020.599623 (PMC7856150; doi:10.3389/fimmu.2020.599623)
Supplement: Supplementary file 4 [file Table_2.doc]

**Supplementary Table 2.** Purity and viability of monocytes and dendritic cells.

|  | Monocytes | | DC viability | | | | |
| --- | --- | --- | --- | --- | --- | --- | --- |
| **Donor** | **Viability** | **Purity** | **iDC** | **mDC** | **mDC TT** | **vitDC** | **vitDC TT** |
| HD1 | 99,3% | 99,1% | 92,5% | 93,5% | 93,9% | 89,6% | 93,8% |
| HD2 | 99,1% | 98,4% | 98,4% | 97,7% | 97,9% | 92,9% | 94,1% |
| HD3 | 99,9% | 92,4% | 98,0% | 97,2% | 98,1% | 96,0% | 96,0% |
| HD4 | 99,4% | 96,7% | 99,0% | 98,8% | 97,5% | 95,2% | 96,8% |
| HD5 | 96,0% | 94,5% | 96,2% | 97,6% | 96,0% | 93,8% | 95,4% |
| HD6 | 99,8% | 97,9% | 94,5% | 88,5% | 90,5% | 87,5% | 86,5% |
| HD7 | 99,2% | 95,5% | 94,7% | 96,3% | 97,5% | 93,9% | 94,1% |
| HD8 | 99,9% | 94,3% | 99,1% | 98,2% | 99,4% | 97,4% | 96,3% |
| HD9 | 99,9% | 89,5% | 96,1% | 95,4% | 92,2% | 95,8% | 92,5% |
| HD10 | 99,9% | 92,9% | 93,8% | 93,8% | 92,9% | 92,1% | 90,5% |
| HD11 | 99,9% | 90,0% | 86,7% | 95,5% | 97,3% | 91,9% | 90,1% |
| HD12 | 99,9% | 94,8% | 91,6% | 95,4% | 94,4% | 90,7% | 93,3% |
| HD13 | 99,9% | 95,4% | 93,1% | 96,4% | 95,6% | 95,2% | 96,5% |
| HD14 | 99,0% | 91,3% | 83,9% | 94,7% | 95,1% | 92,9% | 92,8% |
| HD15 | 99,0% | 93,1% | 94,2% | 94,8% | 93,8% | 84,7% | 90,7% |
| HD16 | 99,0% | 94,5% | 90,9% | 96,0% | 89,0% | 95,0% | 95,0% |
| **MEAN** | **99,3%** | **94,4%** | **93,9%** | **95,6%** | **95,1%** | **92,8%** | **93,4%** |
